# Social network analysis methods for exploring SARS-CoV-2 contact tracing data

**DOI:** 10.1186/s12874-020-01119-3

**Published:** 2020-09-17

**Authors:** Karikalan Nagarajan, Malaisamy Muniyandi, Bharathidasan Palani, Senthil Sellappan

**Affiliations:** Department of Health Economics, Indian Council of Medical Research- National Institute for Research in Tuberculosis, Chennai, 600031 India

**Keywords:** SARS-CoV-2, Social networks, Contact tracing, India, Infectious diseases, Degree centrality, Betweenness centrality, Components, Heterogeneity, Patients

## Abstract

**Background:**

Contact tracing data of severe acute respiratory syndrome coronavirus *2* (SARS-CoV-2) pandemic is used to estimate basic epidemiological parameters. Contact tracing data could also be potentially used for assessing the heterogeneity of transmission at the individual patient level. Characterization of individuals based on different levels of infectiousness could better inform the contact tracing interventions at field levels.

**Methods:**

Standard social network analysis methods used for exploring infectious disease transmission dynamics was employed to analyze contact tracing data of 1959 diagnosed SARS-CoV-2 patients from a large state of India. Relational network data set with diagnosed patients as “nodes” and their epidemiological contact as “edges” was created. Directed network perspective was utilized in which directionality of infection emanated from a “source patient” towards a “target patient”. Network measures of “ *degree centrality*” and “*betweenness centrality*” were calculated to identify influential patients in the transmission of infection. Components analysis was conducted to identify patients connected as sub- groups. Descriptive statistics was used to summarise network measures and percentile ranks were used to categorize influencers.

**Results:**

Out-degree centrality measures identified that of the total 1959 patients, 11.27% (221) patients have acted as a source of infection to 40.19% (787) other patients. Among these source patients, 0.65% (12) patients had a higher out-degree centrality (> = 10) and have collectively infected 37.61% (296 of 787), secondary patients. Betweenness centrality measures highlighted that 7.50% (93) patients had a non-zero betweenness (range 0.5 to 135) and thus have bridged the transmission between other patients. Network component analysis identified nineteen connected components comprising of influential patient’s which have overall accounted for 26.95% of total patients (1959) and 68.74% of epidemiological contacts in the network.

**Conclusions:**

Social network analysis method for SARS-CoV-2 contact tracing data would be of use in measuring individual patient level variations in disease transmission. The network metrics identified individual patients and patient components who have disproportionately contributed to transmission. The network measures and graphical tools could complement the existing contact tracing indicators and could help improve the contact tracing activities.

## Background

Studies concerning the transmission dynamics of SARS-CoV-2 have relied on contact tracing data to estimate the basic reproduction number (R0) for projecting the anticipated number of secondary cases [1, 2]. In addition to its epidemiological importance, contact tracing data is used in intervention settings to contain the spread of SARS-CoV-2 transmission effectively [3]. Contact tracing data helps to identify the possible source of infection, linking exposed individuals and prioritize them for testing and isolation. Outcomes of SARS-CoV-2 contact tracing are primarily reported in terms of case yield and secondary attack rates and sometimes have helped to identify and describe super spreading events [4]. SARS-CoV-2 contact tracing data could also be effectively used for understanding the heterogeneity in disease transmission, especially at the individual patient level [5, 6]. While population level estimates of R0 aims to comprehend the transmission dynamics as a single comprehensive index, individual-level variations in transmission could offer better insights for strengthening prevention interventions [5].

Mathematical modelling methods are mostly adopted for assessing population-level heterogeneity of SARS-CoV-2 infections. Modelling studies have estimated the heterogeneity in terms of over-dispersion levels and proportional variations in transmission based on simulations and assumptions [7–10]. In this background, attempts to measure individual patient-level heterogeneity in SARS-CoV-2 context using real world data could be of direct public health relevance. Characterization of individuals based on different levels of infectiousness could guide the contact tracing interventions to prioritize contact screening, testing and monitoring at in a targeted manner at field level.

While SARS-CoV-2 contact tracing data is continuously collected from hundreds of thousands of SARS-CoV-2 patients, it has not been explored for understanding patient-level heterogeneity using suitable analysis methods. While standard epidemiological analysis methods are in place to generate key parameters of the pandemic, still the suitability of social network analysis methods to assess the transmission heterogeneity has not been attempted partly due to lack of appropriate contact tracing data. Assessing the individual level of variations would require a relational dataset indicative of all contact ties which have occurred between patients and their contacts.

Experiences from past have highlighted the utility of social network analysis methods in successfully exploring individual-level transmission events of infectious diseases like Severe Acute Respiratory Syndrome(SARS), Tuberculosis (TB) and sexually transmitted infections (STIs) [11–15]. Network analysis methods involving quantitative metrics and sociograms could be appropriate and would help to easily comprehend the transmission events at the granular level, i.e. individuals [16, 17]. In this background, we propose to adopt social network analysis methods to assess and understand the heterogeneity of SARS-CoV2 transmission at individual patients level.

## Methods

We utilized standard social network analysis methods to exploratively assess the contact tracing data of SARS- CoV-2 patients in the Indian state of Karnataka to identify the heterogeneity of transmission at the individual patient level. This publicly available contact tracing data of 1959 diagnosed SARS-CoV-2 patients between March 09, 2020, to May 23, 2020, has been collected, compiled and updated by the Department of Health and Family Welfare Services, Government of Karnataka, and other stakeholders for the benefit of the larger public [18–20]. The study data is unique, which represents a cohort of diagnosed patients and contacts who were identified subsequently for a continuous period (75 days). During this period, contact tracing was intensively implemented in the state of Karnataka, which had relatively yielded a higher number of contacts per index patient when compared to other settings in India [21]. Contact information and transmission which had occurred after this period was not available at the time of this analysis. This data is also unique that all patient and contact were linked using unique identifiers which enabled the analysis of data from a relational dataset perspective using social network analysis methods.

Patient’s age, sex, diagnosed date and the epidemiological contact relationships identified through contact tracing efforts were thus readily available in the source data set. A relational network data set was created, with diagnosed patients as *“nodes”* and the epidemiological contact between them as *“edges”.* Each patient was provided a unique identification number (ID), and each patient’s unique epidemiological contact with each other patients was also identified and labelled. The relational data was analyzed from a directed network perspective in which directionality of infection emanated from a “source patient” towards secondary or “target patient”.

Our analysis included only individually identifiable patient-level contacts, and scenarios where such individual identification could not be ascertained (eg common places and travel) were not considered for analysis. This was necessary considering the specific objective of the study to assess the variations at individual patient levels.

### Social network measures

**Fig. 1:**
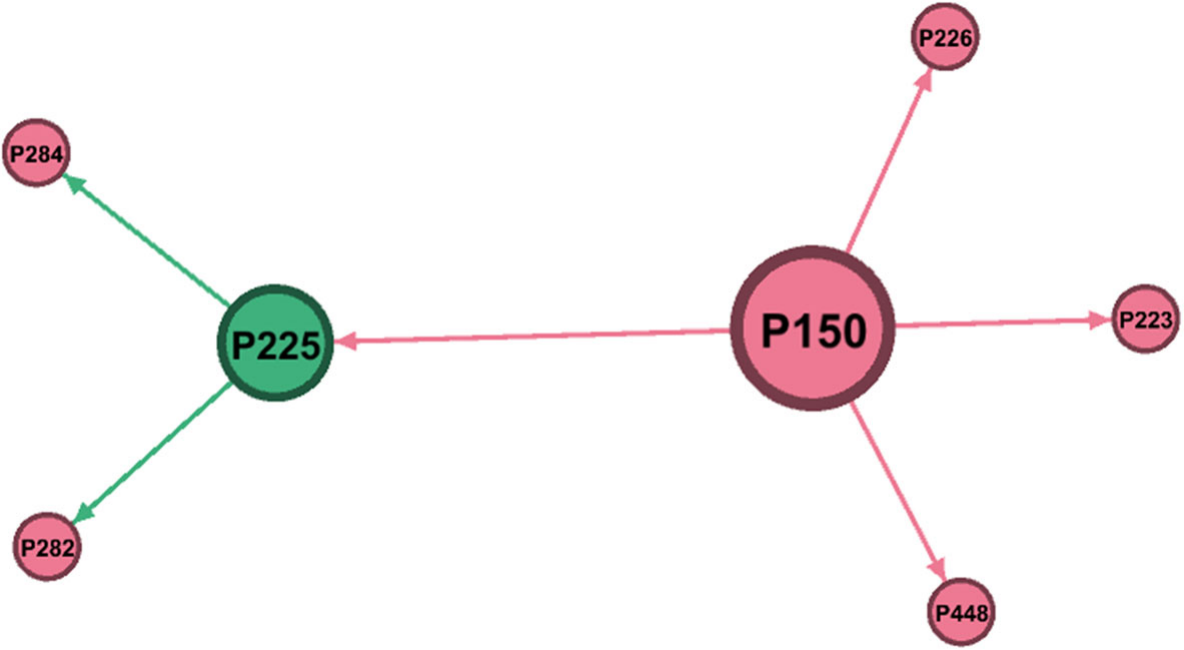
Graphical illustration of network measures. A network of 7 patients denoted by round nodes and the epidemiological relations between them denoted as directed lines with arrows Patient P150 has an out-degree of 4 and have acted as a source of infection to 4 patients P226, P223, P448, P225. Similarly, patient P225 has an out-degree of 2 and had acted as a source patient for P282 and P284. Patient P225 has an in-degree of one and had acted as a target patient who received the infection from P150. Patient P225 depicted as the green node is the only node in the network which has betweenness centrality of 2 and had bridged the transmission from P150 to two other patient P284 and P282 Other nodes is depicted in purple have zero betweenness. The path length between patient P150 and P225 is one m path length from 150 to P282 and P284 is 2. The maximum path length in the network (i.e. network diameter) is also two which lies between P150 and P284, P282. The number of network component for the depicted network is one as all patient s nodes are connected either directly or indirectly to each other, and no patients node is left unconnected

The present analysis calculated social network centrality measures to identify the key nodal patients who were influential in transmitting the SARS-CoV-2 infection. Components analysis was conducted to identify patient sub-network structures which have disproportionately contributed to infection transmission. Average path length and network diameter measures were calculated to assess the dispersion level of infection within patient networks. A graphical explanation of the following social metrics are provided in Fig. 1.

### Measure definitions

**Out-Degree Centrality** measures the number of epidemiological contacts emanating from a patient (node) that are directed to other patients (nodes) in the network. A “zero” out-degree centrality measure for a patient (node) implies that the patient had not transmitted infection to anyone and considered not to be a source patient. A “non-zero” or higher out-degree centrality indicates that the patient is a source of infection for one or many other patients in the network [22, 23]. (Fig. 1).

**In-degree centrality** measures the number of epidemiological contacts incident upon a patient (node). A “zero” in-degree centrality measure for a patient (node) implies that the patient has not been infected by any other patient in the network and thus lack a source of infection. A “non-zero” or higher in-degree centrality indicates that the patient is a target patient who had got the infection from one or more source patient [22, 23]. (Fig. 1).

**Betweenness centrality** measure indicates how frequently a given patient (node) acted as a bridge between other patients (nodes) in the network. This measure is calculated by identifying the shortest contact paths between the patients and further measuring the number of times each patient is found in those shortest paths. A non-zero high betweenness centrality for a patient suggests that the patient had “bridged” infection between a source and target patient. The higher the betweenness centrality, the higher would be the potential of a case to transmit the infection from source patient [24]. (Fig. 1).

**Network component** are connected structures comprising of source and target patients who have epidemiological contacts between them but do not connect to other similar patient components. The component with the largest number of source and target cases was identified as a giant component [25]. (Fig. 1).

**Path length,** in any given network, is the number of edges (relational ties) that is present between a given pair of nodes. The mean path length is the “average of the shortest path length, averaged over all pairs of nodes” in a network. In the present analysis context, it is the shortest path between every source and target patient. The average denotes here the average epidemiological contact distance between the source and target patient. Network diameter denotes the farthest epidemiological contact distance between the source and target patient in the network [26, 27]. (Fig. 1).

### Analysis process

The relational data with nodes and edges were created in Microsoft -Excel software and was further imported into Gephi software (Version 0.9.2) for network analysis [28, 29]. Network measures and components were generated, and the outputs were derived as a data table and sociograms (Network graphs). The network measure tables from Gephi were exported to STATA 15.1 for generating network summary measures for each patient and for calculating percentile ranks. Network graphs or sociograms were generated to visualize relationships patterns between source and target patients. Force atlas layout and Fruchterman Reingold layout in Gephi software were used to visualize patient’s networks [30, 31].

## Results

Of the total 1959 patients, 1203 (61.40%) were male, and 409 (20.87%) were aged 18 or fewer years.

The calculated network measures showed that 1738 (88.72%) patients had an out-degree centrality measure of zero and thus have not acted as a source of infection to other patients. The remaining 221 (11.21%) of patients were found to have a non-zero out-degree measure (range 1–45) and found to be the source of infection to 787(40.19%) other patients in the network. Among these source patients, 0.65% (12) patients have acted as a source of infection to 10 or more target patients and have collectively infected 37.61% (296 of 787) target, patients. (Table 1).

**Table 1:** Source patient categorization based on out-degree centrality measures (*n* = 1959)

Out-Degree centrality measure	No of patients	Per cent	Percentile Rank	Source patient status
0.00	1738.00	88.72	44.35	No
1.00	96.00	4.90	91.16	Yes
2.00	39.00	1.99	94.61	Yes
3.00	30.00	1.53	96.37	Yes
4.00	18.00	0.92	97.60	Yes
5.00	12.00	0.61	98.36	Yes
6.00	1.00	0.05	98.69	Yes
7.00	7.00	0.36	98.90	Yes
8.00	5.00	0.26	99.20	Yes
10.00	1.00	0.05	99.36	Yes
11.00	2.00	0.10	99.43	Yes
12.00	1.00	0.05	99.51	Yes
15.00	1.00	0.05	99.56	Yes
17.00	1.00	0.05	99.61	Yes
19.00	1.00	0.05	99.66	Yes
26.00	1.00	0.05	99.71	Yes
29.00	1.00	0.05	99.77	Yes
31.00	1.00	0.05	99.82	Yes
34.00	1.00	0.05	99.87	Yes
36.00	1.00	0.05	99.92	Yes
45.00	1.00	0.05	99.97	Yes

Of the total 1959 patients, 1218(62.17%) patients had an in-degree centrality measure of zero and thus lacked any epidemiological contact with a source patient. Of the remaining, 707(36.09%) of patients had an in-degree measure of one, implying that one source patient was identified for each of them. A minimal number of patients 34 (1.73%) had an in- degree of more than one (range 2–5) implying more than one source patient were identified for each of them. (Table 2) Degree centrality measures summarized that a total of 787 epidemiological contacts occurred between 221 source patients and 741 target patients.

**Table 2:** Target patient categorization based on In-degree centrality measures (*n* = 1959)

In-degree centrality measure	No of patients	Per cent	Percentile Rank	Target patient status
0	1218	62.17	47.62	No
1	707	36.09	80.21	Yes
2	29	1.48	99	Yes
3	1	0.05	99.77	Yes
4	1	0.05	99.82	Yes
5	3	0.15	99.92	Yes

Betweenness centrality measures was zero for 1886 (95.25%) of patients implying that they were not having any bridging role in the transmission of infection between any other pair of patients. The remaining 93 (7.50) patients had a non-zero betweenness centrality measure (range 0.5 to 135) implying their bridging in transmission between other patients. (Table 3) The summary measure of centrality measures shows the highly skewed distribution of the out-degree and betweenness centrality measures. (Table 4).

**Table 3:** Bridging role categorization of patients by betweenness centrality measure (*n* = 1959)

Betweenness centrality measure	No of cases	Per cent	Percentile Rank	Bridging role in the transmission
0	1866	95.25	47.62	No
0.5	1	0.05	95.27	Yes
1	27	1.38	95.99	Yes
1.5	1	0.05	96.7	Yes
2	17	0.87	97.16	Yes
2.5	1	0.05	97.62	Yes
2.5	1	0.05	97.67	Yes
3	12	0.61	98	Yes
3.25	1	0.05	98.34	Yes
3.5	1	0.05	98.39	Yes
4	2	0.1	98.46	Yes
4.75	1	0.05	98.54	Yes
5	1	0.05	98.59	Yes
6	5	0.26	98.74	Yes
6.08	1	0.05	98.9	Yes
7	2	0.1	98.97	Yes
8.33	1	0.05	99.05	Yes
9	2	0.1	99.13	Yes
10	5	0.26	99.31	Yes
11	2	0.1	99.48	Yes
12	1	0.05	99.56	Yes
15	1	0.05	99.61	Yes
19	2	0.1	99.69	Yes
20	1	0.05	99.77	Yes
32	1	0.05	99.82	Yes
47	1	0.05	99.87	Yes
92	1	0.05	99.92	Yes
135	1	0.05	99.97	Yes

**Table 4:** Summary statistics of network centrality measures

Summary statistics	Out-degree centrality measure of patients (nodes)	Betweenness centrality measure of patients (nodes)	In degree centrality measure of patients (nodes)
**Mean**	0.72	0.34	*0.4*
**SD**	2.23	4.09	0.55
**Variance**	5	16.74	*0.3*
**Skewness**	12.14	25.2	1.61
**Kurtosis**	186.19	738.96	9.92

**Fig. 2:**
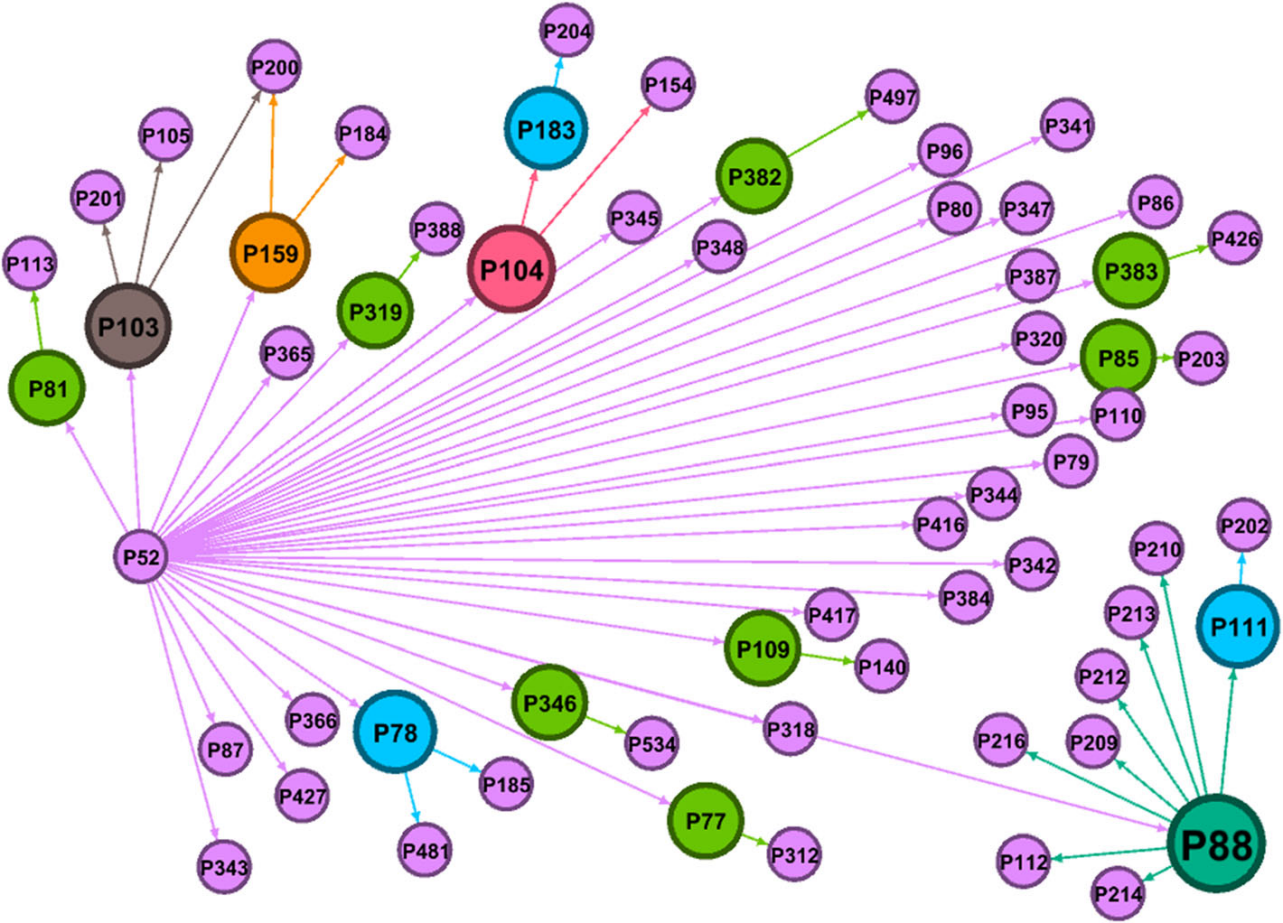
Graphical representation of C1, a giant component with 63 (3.22%) patients and 63 (8.01) transmission contacts. Giant component represented in the sociogram consists of 63(9.35% of 673) cases (circular nodes) and 63 (12.96% of 486) epidemiological contacts (edges denoted by arrowed lines). The size of a node is proportional to its out-degree centrality. The arrows denote the direction of infection from source to target cases. Patient P52 has the largest out-degree (36) and acted as a source case for 36 target cases. The next influential node was P88, who acted as the source case for nine target cases. Other influential source cases were P104, P 78, and P159 who had out-degree of 2. Nodes P111, P183, P77, P81, P 85, P109, P319, P346, P382, and P383 were the source case for one target case each. The colour of the node indicates the category of betweenness centrality measure. Lavender colour denotes, zero betweenness, leaf green nodes denote betweenness of one, blue denotes betweenness of 2, saffron denotes betweenness of 1.5, grey denotes betweenness of 2.5, rose denotes betweenness of 3, dark green denotes betweenness of 9 for P88 which acted as a bridge of transmission between source case P52 and target cases P112, P210, P216, P212, P214, P209, P212, P111 and P202. The path length between P52 and P202 is 4, which was the maximum and thus the diameter of the network. P52 had in-Degree of zero, P200 had in-degree of 2, and all other patients had one in-degree

A total of 1207 sub network components were identified, which together comprised 1959 patients with and without epidemiological contacts. Of these 1207 components, nineteen components with at least ten patients and ten contact relationships were identified, which overall accounted for 26.95% (529) of the total patients (1959) and 68.74% (541) of total transmission contacts (787) of the network. These nineteen components have each proportionately represented 3.22 to 0.5% of total patients and occurred within a time interval of 13 to 48 days. Components were not occurring consecutively but mostly simultaneously over time. (Table 5) The largest patient component had 63 (3.22%) patients and 63 (8.01) transmission contacts. **(**Fig. 2).

**Table 5:** Network components of 1959 SARS-CoV-2 diagnosed patients

Patient components	No & % of patients in components	No & % of transmission contacts in components	components initiation date^a^	components end date^a^	Cycle^b^	Patients with Out-degree centrality > = 97.5th percentile	Patients with Betweenness centrality > =97.5th percentile
C1	63 (3.22)	63 (8.01)	Mar 262,020	Apr 292,020	34	P52,P88	P88, P103,P104
C2	49 (2.50)	48 (6.10)	Apr 092020	May 192,020	40	P653	P208,P420, P653
C3	46 (2.35)	51 (6.48)	Apr 032020	May 212,020	48	P128,P224,P483,P496,P547	P224,P483, P484,P486, P496,P539,P547,P552,P721
C4	41 (2.09)	41 (5.21)	Apr 122,020	May 172,020	35	P221,P306	P306,P362,P 510
C5	40 (2.04)	39 (4.96)	Apr 292,020	May 212,020	22	P533,P651	P651
C6	37 (1.89)	36 (4.57)	Apr 302,020	May 212,020	21	P556,P581,P662	P581,P662P,P667,P852
C7	35 (1.79)	34 (4.32)	Apr 222,020	May 092020	17	P419	–
C8	31 (1.58)	30 (3.81)	May 052020	May 182,020	13	P659	–
C9	28 (1.43)	27 (3.43)	Apr 102,020	May 122,020	32	P205,P425	P395,P425,P 515,P529,P 532
C10	25 (1.28)	35 (4.45)	Apr 042020	May 142,020	40	P134,P138,P171,P179	P179,P171, P371
C11	22 (1.12)	21 (2.67)	May 032020	May 192,020	16	P607	0
C12	18 (0.92)	18 (2.29)	Apr 192,020	May 132,020	24	P507,P536	P432,P501, P507,P536,P578
C13	17 (0.87)	23 (2.92)	Apr 072020	May 032020	26	P167,P168	P350
C14	15 (0.77)	18 (2.29)	Apr 032020	May 052020	32	P125,P165,P186	P165,P186, P367,P368
C15	14 (0.71)	13 (1.65)	Apr 282,020	May 232,020	25	P913	P913
C16	13 (0.66)	12 (1.52)	Apr 052020	May 182,020	43	P245	P245,P301,P575
C17	13 (0.66)	12 (1.52)	May 022020	May 202,020	18	P590	–
C18	11 (0.56)	10 (1.27)	Mar 312,020	Apr 172,020	17	P141	–
C19	10 (0.51)	10 (1.27)	Mar 212,020	Apr 092020	19	P19	–
**C1-C19 Summary**	**528 (26.95%)**	**541 (68.74%)**	**21-March to May 232,020**		**63 days cycle**	**36 patients**	**45 patients**

^a^Cycle refers to the time period between the first and last diagnosed case of the **components**; ^b^ **Component** initiation date is the earliest diagnosis date of a case in that **components** and **components** end is the latest date of diagnosis of a case in that **components**

The membership of these nineteen patient components showed that they were disproportionately represented by patients who had highly skewed out-degree and betweenness centrality measures. Of all 57 patients with the highest out-degree centrality (ranking 97.5 and above), 38(64.28%) were represented in these nineteen patient components. Similarly of all 47 patients with the highest betweenness centrality (ranking 97.5 and above), 45 (95.74%) of them were represented in these nineteen patient components. (Table 5).

The mean path length between the patients was measured as 1.53, and the network diameter (or the largest path length between patients) was measured as 4. **(**Fig. 2**).**

## Discussion

Epidemiological studies on SARS-CoV-2 have emphasized the importance of heterogeneity in transmission and the need for measuring transmission events and variations at the individual levels [7–10]. These studies have emphasized the importance of heterogeneity information in addition to estimating basic reproduction number. Through this study we have attempted to adopt the standard social network analysis methods to measure heterogeneity by analyzing a large scale contact tracing data of SARS-CoV-2. Availability of relational dataset consisting of uniquely identified and linked patient and contacts was an advantage which enabled the adoption of network analytical methods.

Our adopted network analysis methods highlights the individual patient level variation in SARS-CoV-2 transmission. The out-degree centrality measures highlight that while the majority (88.72%) of the patients in the cohort had not transmitted infection, only a minuscule proportion of source patients (0.65%) have disproportionately transmitted the infection to 36.7% of target patients. In-degree, centrality measures shows that as much as 62.17% of diagnosed patients did not have any source of infection and less proportion of patients (1.72%) had more than one source of infection. Betweenness centrality measures highlights that not all infections were directly transmitted from few influential source patients (with higher out-degree) to many target patients, but only through influential bridging patients (with high betweenness) who transmitted the infection from them.

The centrality measures were thus able to identify and measure the differences among individual patients to infect others, getting infected and being able to transmit the infection as an intermediary to many others. The summary statistics of the out-degree and betweenness measures hints a heavy-tailed distribution with only a few actors in the network playing an influential role in being source and intermediaries of transmission. However, we have not attempted to prove the network distributional properties in this study since that would require a comprehensive social network survey data collected from SARS-CoV-2 patients and their contacts. The contact tracing data used by us do not have information on symptom onset, confirmation and related information which would be required for such analysis.

The network components analysis identified 19 key components which have contributed to almost two-thirds of the total transmission events between patient and contacts. Our findings show that the maximum transmission had happened within these nineteen components, which each proportionality represented only 3.22 to 0.5% of the total patients. The component analysis showed that source patients with high out-degree had passed their infection mostly through various intermediaries i.e. those with high betweenness. For example, in the identified giant component with 63 patients, **(**Fig. 1**)** it was identified that the first source patient (P52) had not transmitted infection to all 62 target patients by himself but through the considerable number of intermediaries. It was found that these individuals with high out-degrees and betweenness, (who could be called influencers) are predominantly found in these 19 components with have contributed to 68.7% of transmission. From this, we could hypothesize that influential patients as small connected components may contribute to much of the transmission events when compared to sole influencers or super spreaders [32–34].

The path length metrics indicated that on average any source and target patients in the network could be reached by crossing 1.5 steps and at a maximum, it was maximum four steps between the farthest placed source and target patients. This highlights that the transmission of infection is not widely dispersed through multiple waves from source patient and is limited to less than two waves on average.

Our application of network analysis methods holds importance from two aspects. First, from a methodological perspective, our proposed network analysis methods could be easily adapted for large scale contact tracing data for SARS-CoV-2. However, it also necessitates the availability of a properly line listed and linked patient –contact data. While we have used considerably large data available during the initial months of the pandemic, the availability of such data is only rare in most settings [35]. Thus our proposed methods also implicitly recommend for continuous collection and systematic management of contact tracing data using unique identification numbers (IDs) for all patients and their identified contacts.

Secondly, the adopted network analysis methods and the measures could be of importance for public health implementers who are directly involved in the contact tracing data of SARS-patients. At present SARS-CoV-2 contact tracing data is systematically collected only in few settings and is analyzed to summarise population and large cluster level dynamics and heterogeneity levels [36]. Our adopted network analysis methods could complement such efforts and help to precisely characterize all individual-level variations in transmission at source and target patient levels. While we have adopted network analysis retrospectively, prospective identification of influential patients (with high out-degree and betweenness measure) and closely connected patient groups (components) could enable prioritizing the contact tracing activities in a more targeted way. Individual patients who have disproportionately infected others could be followed up, and their contacts could be intensely monitored for interrupting transmission and facilitate early diagnosis. For examples, in our analysis, the largest patient component (C1) with 63 patients, patient P52 had the largest out-degree centrality measure of thirty six. **(**Fig. 1**)** If network analysis had been used to analyze the emerging data at that time concurrently, the out-degree measure of P52 patient could have signalled him as an influential source patient at an early time and could have enabled targeted and intense monitoring of all the contacts. Considering the wider time duration (63 days on average) (Table 5) that elapsed between the first and last diagnosed patient in the nineteen key components which contributed to majority of infections, network measures could had been of use to fast track the contacts of influential patients in these components.

The network measures and components could thus help the public health personnel to comprehend the key influential patients and patient groups (components) in their intervention settings. Basic network measures could be of importance for public health planners who would have to deal with contact tracing data of tens of thousands of patients on a daily basis and comprehend it. The quantitative measures and graphical tools of network method could be useful to analyze exhaustively and easily interpret the large scale contact tracing data which otherwise remain underutilized. We understand that contact tracing data on SARS-CoV-2 holds more valuable information and fine details which could be best explored by adopting complementary methods like social network methods, which has been successfully used in other infectious diseases contexts [11–15].

## Conclusion

Our adopted social network analysis approach was found useful in capturing the heterogeneity of SARS-COV-2 transmission at the individual patient level by analyzing the contact tracing data from a network perspective. The method had helped identify the key individual patients and components which could help the public health implementers to focus their contact tracing activities. The network measures and graphical tools could complement the existing contact tracing indicators. Prospective adoption of network analysis could help explore large volumes of contact tracing data to detect heterogeneity and thus could aid implementing contact tracing activities in a better-informed manner.

## Data Availability

The data used for the study were obtained from the daily media bulletins of the Department of Health and Family Welfare Services, Government of Karnataka, India published online through URL https://covid19.karnataka.gov.in/govt_bulletin/en . The synthesised data was obtained from online source at https://en.wikipedia.org/wiki/COVID-19_pandemic_in_Karnataka. The latest updated data could be found at the data repository of online at https://www.isibang.ac.in/~athreya/incovid19/data.html .The raw data and structured network relational data are attached to the manuscript as supplementary files. The codes and techniques used for network analysis and descriptive analysis are available from the corresponding author on reasonable request.

## Abbreviations

SARS: Severe Acute Respiratory Syndrome
SARS-CoV-2: Severe Acute Respiratory Syndrome Coronavirus 2
R0: Basic reproduction number
TB: Tuberculosis
STI: Sexually Transmitted Infections
ID: Identification number (ID)
SARS: Severe Acute Respiratory syndrome
MERS: Middle East Respiratory Syndrome
